# On the Machinability Evolution in Asymmetric Milling of TC25 Ti Alloy Aiming at High Performance Machining

**DOI:** 10.3390/ma14237306

**Published:** 2021-11-29

**Authors:** Xueli Song, Hongshan Zhang

**Affiliations:** 1 School of Mechanical and Automotive Engineering, Qilu University of Technology (Shandong Academy of Sciences), Jinan 250353, China; 2 Shandong Institute of Mechanical Design and Research, Jinan 250031, China; 3 Jinan Cigarettes Factory, China Tobacco Shandong Industrial Co., Ltd., Jinan 250104, China; zhang_hongshan@163.com

**Keywords:** machinability, asymmetric up-milling, TC 25 alloy, high performance machining

## Abstract

In this paper, the evolutions of cutting force, cutting temperature, and surface roughness, and the corresponding machinability in asymmetric up-milling of TC25 alloy are investigated. The results indicated that radial depth of cut generated opposite influence on the cutting force/cutting temperature versus surface roughness. The reason can be accounted as the intertwining of feed marks at low radial depth of cut, and the mechanism of hard cutting at a high radial depth of cut. Moreover, the asymmetry has a significant effect on the machinability in asymmetry up-milling TC25 alloy. Changing the asymmetry, i.e., the radial depth of cut, can alter the machinability while maintain the balanced development of various indexes. The machinability reaches the best when the radial depth of cut is *a_e_* = 8 mm. The axial depth of cut and feed per tooth should be selected as large as possible to avoid work hardening and to improve machining efficiency in asymmetric up-milling TC25 alloy. The cutting speed should be controlled within *V_c_* = 100–120 m/min to obtain better machinability. On the basis of this research, it is expected to find optimized milling parameters to realize high efficiency milling of TC25 alloy.

## 1. Introduction

The contradiction between machining efficiency and quality is the most prominent contradiction in today’s manufacturing industry. Research shows that we must continue to pursue the improvement of efficiency if we want to fully and effectively deal with other contradictions in the manufacturing industry. Therefore, machining efficiency is pushed to a crucial position in the development of today’s manufacturing industry. High performance machining has gradually developed into an inevitable trend of modern manufacturing technology [1,2].

TC25 alloy belongs to the Ti–A1–Sn–Zr–Mo–W–Si series of titanium alloys, which is an (α + β) typed heat-strength titanium alloy with good comprehensive properties. TC25 alloy also has the high thermal stability of titanium alloys BT8 and BT9. It can work for a long time in a working environment of 500~550 °C, which is an ideal metal material for aero engines and a common material for compressor parts on engines. However, literatures [3,4] mainly studied the microstructure and fatigue performance of TC25 alloy, while few literatures [5] on its cutting performance, meaning there is a lack of guidance for the highly efficient machining of TC25.

There are mainly the following two problems in the actual cutting process of titanium alloys. One is the relative low machining efficiency. At present, the cutting of titanium alloys becomes very difficult when cutting speeds are over 30 m/min using high-speed steel cutting tools or over 60 m/min by using cemented carbide cutting tools [6]. The cutting speed in the high-speed cutting of titanium alloys is generally above 100 m/min, so the cutting efficiency is lower in the case of high-speed cutting [7]. However, the cutting speed in the high-speed milling of titanium alloy at abroad is as high as 100~200 m/min, and the application range of high-speed cutting of titanium alloy is relatively wide. Domestically speaking, the current cutting speed of titanium alloys ranges from 30 to 50 m/min, which is a big gap when comparing with foreign countries.

The second is the relatively poor surface quality because of the serious tool wear progression. The reason accounts that the low thermal conductivity of titanium alloy hinders the heat loss during the machining process. As known to all, little heat is given off by the chip and most is absorbed by the cutting tool in cutting of titanium alloys, i.e., adequate heat dissipation, which is one of the biggest problems during machining. As a result, the heat generated during cutting is mainly concentrated at the cutting edge of the cutting tool, which accelerates the wear of the cutting tool and deteriorates the surface quality of the machined surface. For this reason, scholars commonly carried out works on tool wear and surface roughness to get the relations with the cutting temperature [8,9]. Additionally, the cutting temperature must be one of the factors to evaluate the machinability of titanium alloys.

At present, more and more researchers focused on the high-speed and high efficiency cutting of Ti alloys. High-speed and high-efficiency cutting has the advantages of high production efficiency, good product quality, and the ability to process thin-walled parts. It can effectively reduce surface roughness and cutting force, slow down the increase trend of cutting temperature, and, in turn, reduce thermal deformation.

Yang et al. [10] found that it was prone to produce saw-tooth-shaped cutting chips during high-speed turning of titanium alloys, resulting in high-frequency periodic fluctuations in cutting force. In addition, the low deformation coefficient and thermal conductivity of titanium alloys resulted in large specific cutting force, high cutting temperature, and serious spring-back in high-speed machining of titanium alloys, which could aggravate the progression of tool wear, and seriously deteriorate the surface integrity of the machined surface. Yang et al. [10] concluded that using a low cutting speed, large feed per tooth, and a large cutting depth could reduce the generation of saw-tooth-shaped cutting chips. Ma et al. [11] experimentally analyzed the stability of chip flow in cutting Ti6Al4V alloy. It was observed that the chip morphology transformed from continuous to saw-tooth-shaped and then serrated cutting chips with the increase of cutting speed. The above research fully shows that titanium alloy is a typical difficult-to-cut material, and its machinability must be improved. Gupta et al. [12] found that the machining performance of Ti6Al4V in the turning process can be enhanced by applying cryogenic cooling environments. Pimenov et al. [13] further reviewed the application of cooling-lubrication techniques in order to improve the machinability of Ti and its alloys. However, the use of dry cutting is more conducive to achieving sustainability and clean manufacturing.

The cutting parameters have a very significant effect on cutting force and cutting temperature. Narutaki [14] studied the influence of cutting speed on both cutting force and cutting temperature through turning of TC4 alloy with cemented carbide cutting tools. The results showed that the cutting speed had no obvious influence on cutting force when turning titanium alloy TC4 with cutting speed *V_c_* = 20–200 m/min, where the cutting force was about 70% of that in turning AISI 45 steel under the same conditions. Hence, cutting force was not the main reason for aggravating tool wear. However, the cutting temperature in the turning of titanium alloy TC4 could reach 700 °C or more. Especially, the cutting temperature in the turning of titanium alloy could be higher than 1000 °C with a cutting speed of *V_c_* = 300 m/min, which was about 1.5 times of that in machining AISI 45 steel under the same conditions. It could be concluded that the poor machinability in the turning of titanium alloy was mainly due to the high cutting temperature as a result of adequate heat dissipation [14]. Dewes et al. [15] experimentally investigated the cutting temperature in high-speed milling die steel by using both the infrared camera method and artificial thermocouple method. Cotterell and Byrne [16] further established a thermal model for the prediction of the formation of saw-tooth-shaped cutting chips during the cutting process of titanium alloy TC4, based on which the average temperature on the rake face and the main shear zone could be predicted.

The surface roughness of the machined surface is another important indicator to characterize the quality of parts. Kumar et al. [17] investigated the effect of cutting parameters (cutting speed, feed rate and the depth of cut) on surface roughness when turning Ti6Al4V titanium alloy with medium temperature chemical vapor deposition (MT-CVD) tool insert. The results indicated that the influence of feed on surface roughness is the greatest, followed by the depth of cut and cutting speed. Oosthuizen et al. [18] studied the effects of cutting speed and feed rate on surface roughness, microhardness and microstructure when milling Ti6Al4V. The surface roughness increases as the feed rate increases and the cutting speed decreases. Sun et al. [19] conducted an end milling test on the titanium alloy TC4 and found that the surface roughness of the machined surface along the feed direction and perpendicular to the feed direction had different evolutional trends. Surface roughness along the feed direction increased first and then decreased with the increase of cutting speed within the range of *V_c_* = 50–110 m/min, while the surface roughness perpendicular to the feed direction showed a decreasing trend. Many scholars had further conducted in-depth research on how to improve the surface quality in machining titanium alloy. Sharman et al. [20] analyzed the effect of cutting speed on surface quality within the range of *V_c_* = 25–40 m/min. The results showed that both the size and density of micro-cracks generated on the machined surface were decreased with high cutting speed, which was benefitted to improve the surface quality of the machined surface.

**Figure 1:**
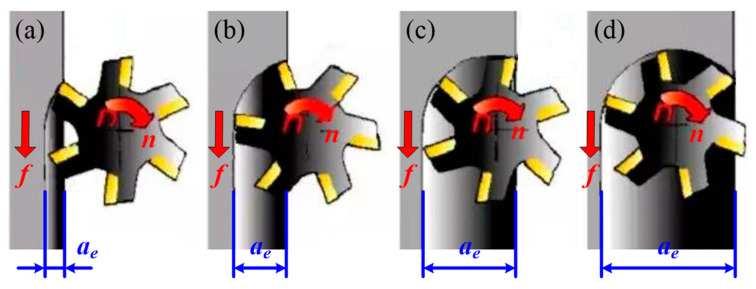
Schematic diagram of asymmetric milling with the radial depth of cut increasing from small (**a**,**b**) to large (**c**,**d**).

Although the machining parameters are considered in the above research, the relative position between the milling tool and workpiece is ignored. As known to all, the milling process can be divided into symmetrical milling and asymmetrical milling based on the different positions between the milling tool and workpiece when milling a plane. With respect to machinability, asymmetric milling is better than symmetric milling [21,22]. Asymmetric milling can be further divided into asymmetric up-milling and asymmetric down-milling. Figure 1a, b present how the pure up-milling as the radial depth of cut is smaller than half of the tool diameter. However, the kinematics of asymmetric up-milling changes into down-milling when the radial depth of cut exceeds half of the tool diameter, as shown in Figure 1c, d.The tool teeth cut into the workpiece from the smallest cutting thickness and is cut out from the largest cutting thickness in asymmetric up-milling, while it is just the opposite asymmetric down-milling. As a result, the tool vibration in asymmetric down-milling is greater than that of asymmetric up-milling, and the gap between the screw and the nut of the workbench should be eliminated to prevent the workbench from moving in the case of large horizontal milling force component. Favero Filho et al. [23] indicated the machining direction (i.e., up- and down-milling) generated a much more significant influence on the machinability when compared to the cutting parameters. The better machinability of up-milling processes in relation to down-milling was also found. Hence, the asymmetric up-milling was utilized for milling TC25.

In our previous work [5], the machinability of TC25 was investigated by comparing the differences in cutting force, cutting temperature and surface roughness between TC25 and TC4 alloys. It is found that TC25 has worse machinability than TC4, that is, larger cutting force, higher cutting temperature and worse surface roughness. Therefore, it is very necessary and urgent to study the influence of machining parameters on the machinability of TC25. In addition, empirical formula models were used to analyze the machinability of TC25 and TC4 in the literature [5]. A more accurate numerical prediction model is needed for investigating the influence of cutting parameters on machinability. In this paper, the evolutions of cutting force, cutting temperature, and surface roughness in asymmetric milling TC25 alloy are investigated based on the Taguchi method and analysis of variance (ANOVA), aiming at exploring the optimized milling parameters for high-efficiency and high-quality milling of TC25 alloy. On the basis of this work, it is expected to provide theoretical guidance for the high-efficiency and high-quality milling of TC25, and then promote the application of TC25 for aero engines and/or compressor parts on engines.

## 2. Experimental Details

**Figure 2:**
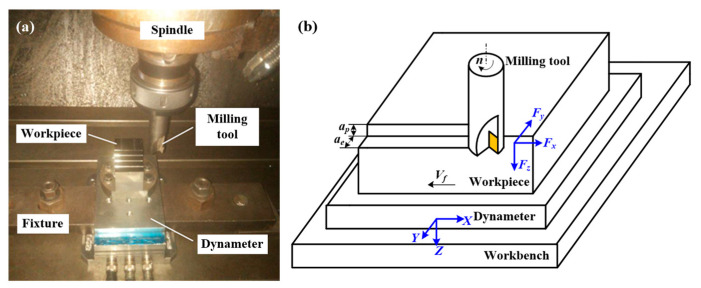
(**a**) Experimental setup and (**b**) illustration of cutting parameters in asymmetry milling.

In this paper, asymmetric up-milling of TC25 alloy were carried out based on Taguchi’s experimental design. The milling experiments were carried out on DAEW00 AVE-V500 machining center, as shown in Figure 2a. The size of the machined surface of the workpiece was 40 × 70 mm, which was stepped after machining to retain the relevant information of the machined surface under each group of cutting parameters. The tool insert used in the experiments was carbide insert XOMX120408TR-ME08 F40M (SECO, Sweden) with (Ti, Al)N-TiN composite coating, which is amounted on the tool holder R217.69-2525.0-12-2AN (SECO, Sweden). The diameter of the tool holder is *Φ*25 mm, and the tool insert is equipped with a rake angle of 25°, relief angle of 14°, nose angle of 80°, and a nose radius of 0.8 mm. Only one insert is installed in order to avoid runout errors during the milling process. During the cutting experiment, a new insert was used for each set of cutting conditions in order to ensure the sharpness of the cutting edge. Additionally, the cutting distance of the cutting tool was very short. With these considerations, the tool wear of the cutting tool could be negligible. The cutting force was measured with the Kistler 9275B Dynameter, cutting temperature of the cutting zone was measured by the TH5104R thermal imaging camera (NEC, Japan), and surface roughness was measured by using the NT9300 optical profiler (Wyko, America). Three repetitions were performed for each trial to ensure the reliability of the results.

The Taguchi method is useful for determining the best combination of factors under desired experimental conditions [24,25,26]. The Taguchi method reduces a large number of experiments that could be required in traditional experiments when the number of process parameters increases. In the Taguchi method, an orthogonal array is designed that studies the entire parameter space with a small number of experiments.

As illustrated in Figure 2b, there are four cutting parameters, i.e., radial depth of cut *a_e_*, axial depth of cut *a_p_*, cutting speed *V_c_* (*V_c_* = *n*π*d*/1000), and feed per tooth *f_z_* (the displacement of the milling tool relative to the workpiece in feed direction when the milling tool rotates an inter-tooth angle in milling with multi-teeth milling tools, and *f_z_* = *V_f_*/*nz*, where *V_f_* represents the feed velocity (mm/min), *n* is the rotating speed of the spindle (r/min), and *z* is the number of teeth of the milling tool), for asymmetric milling. If a full factor experimental design is performed, 256 sets of experiments are required in the case of four values for each variable. The orthogonal experimental design based on the Taguchi method can reduce the number of experiments to 16, which greatly reduces the time as well as the cost of the experiment. The factors and levels, and the orthogonal experimental series L_16_(4^4^) (including experimental results) are shown in Table 1 and Table 2, respectively. In order to make the milling process cover a complete up-milling process, the maximum radial depth of cut is determined slightly larger than half of the tool diameter. After all milling experiments, mintab software was utilized for statistical analysis and model establishment.

## 3. Results and Discussion

### 3.1. The Effect of Cutting Parameters on Cutting Force

**Figure 3:**
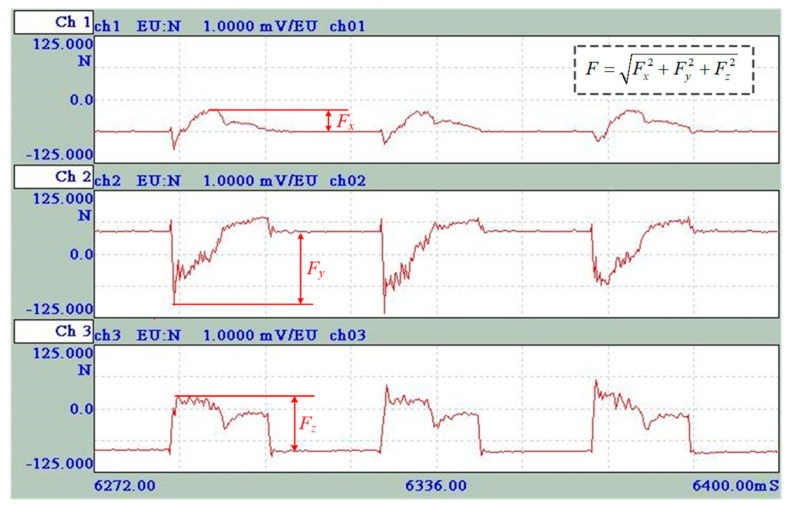
Typical cutting force signal spectrum in asymmetric milling (trial No. 9).

Figure 3 shows the typical cutting force signal spectrum in asymmetric up-milling. Obviously, the horizontal component of cutting force *F_x_* is opposite to the feed direction of the workpiece, which is a characteristic of up-milling. Considering that the up-milling is an interrupted cutting process, in which the uncut chip thickness changes at any time, the cutting force changes periodically and also changes with time within a period. Hence, the maximum cutting force, rather than average cutting force, was utilized in the present study. The resultant cutting force had been widely utilized for the selection of cutting tools and the optimization of cutting parameters in milling hard-to-cut material [27,28]. Each component of cutting force only reflects one aspect of machinability, while the resultant cutting force can fully characterize the machinability. Hence, at least five periods in the cutting force signal spectrum are selected to extract the maximum value of each component of cutting force *F_x_*, *F_y_*, and *F_z_*, and then the average values accordingly are used to calculate the resultant cutting force *F*.

**Figure 4:**
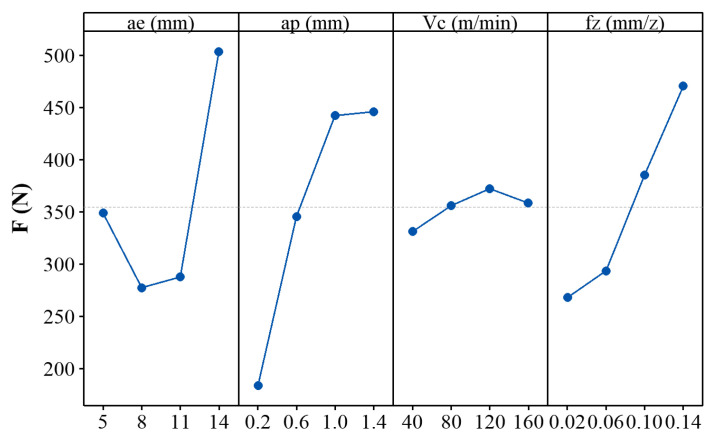
Plot of means for the maximum cutting force *F*.

Figure 4 shows the plot of means for the maximum cutting force *F*. It is found that the radial depth of cut has a significant effect on the cutting force during asymmetric up-milling of TC25 alloy. In addition to radial depth of cut, the axial depth of cut and the feed per tooth also have a significant effect on the cutting force. This is because that the above cutting parameters have a direct effect on the material removal volume, which has a positive correlation with cutting force. At the same time, it was also found that the cutting speed has a slight effect on the cutting force. This is because the influence of the cutting speed on strain rate is much greater than the influence of cutting speed on strain. In this study, the increase/decrease in cutting speed is not sufficient to generate a significant change in the cutting force.

**Figure 5:**
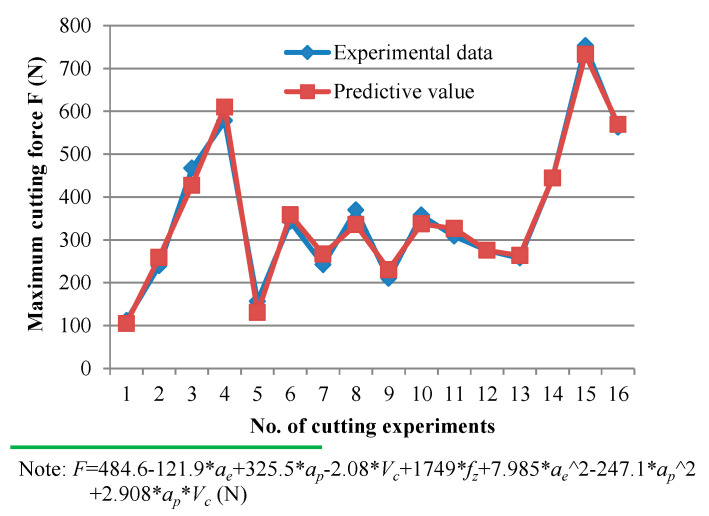
Experimental data [L_16_(4^4^)] and fitting results of maximum cutting force *F*.

The regression equation between the maximum cutting force and machining parameters (*a_e_*, *a_p_*, *V_c_*, and *f_z_*) was established based on a Taguchi analysis. The interaction of three or more factors will be very weak, so it will not be considered here. The method of backward elimination of the coefficients was used for the fitting of a regression model. The fitting results, as shown in Figure 5 (the corresponding ANOVA results are summarized in Table 3), show that the quadratic regression equation has a strong predictability (the *p*-value is far less than *α* = 0.05) with adjusted squared fit factor *R*-sq. (adj) = 96.88%.

**Figure 6:**
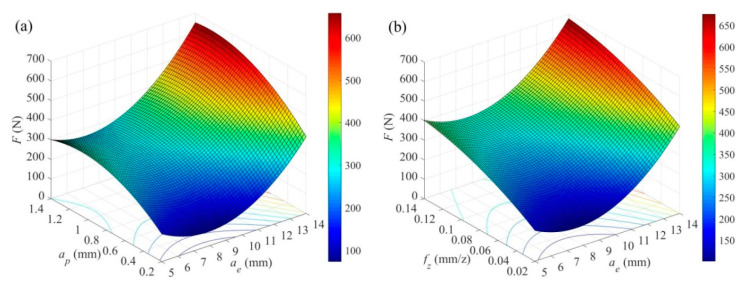
The interaction of (**a**) *a_e_* × *a_p_* (*V_c_* = 80 m/min, *f_z_* = 0.06 mm/z), and (**b**) *a_e_* × *f_z_* (*V_c_* = 80 m/min, *a_p_* = 0.6 mm) on maximum cutting force *F*.

Figure 6a,b show the interaction of radial depth of cut versus axial depth of cut, and radial depth of cut versus feed per tooth on maximum cutting force, respectively. At the same time, other cutting parameters are fixed at the level of −1 to make the machining process relatively mild. It can be seen from Figure 6 that the maximum cutting force first decreases and then increases with the increase of radial depth of cut, reaching a minimum when the radial depth of cut is *a_e_* = 7.5 mm (about 30% of the tool diameter). This is because the radial depth of cut in asymmetric up-milling process determines the tool cutting path trajectory. The tool insert quickly separates from the workpiece after cutting into when the radial depth of cut is small. Under these circumstances, the machining stability is very poor, and as a result, the cutting tool is severely impacted. As the radial depth of cut increases, the cut into and out of the cutting tool becomes stable, and the actual cutting time increases during each revolution of the cutting tool. Hence, the mean cutting force increases. The cutting force continues to increase until it stabilizes as the axial depth of cut and feed per tooth increase. This is because the unit material removal volume increases as the axial depth of cut and feed per tooth increase, which in turn causes the increase in mean cutting force. Therefore, the proper radial depth of cut (approximately 30% of the tool diameter under this experimental conditions), larger axial depth of cut and feed per tooth should be selected in asymmetric up-milling TC25 alloy in order to get low cutting force (which is a benefit for tool life) without losing material removal rate or productivity.

### 3.2. The Effect of Cutting Parameters on Cutting Temperature

**Figure 7:**
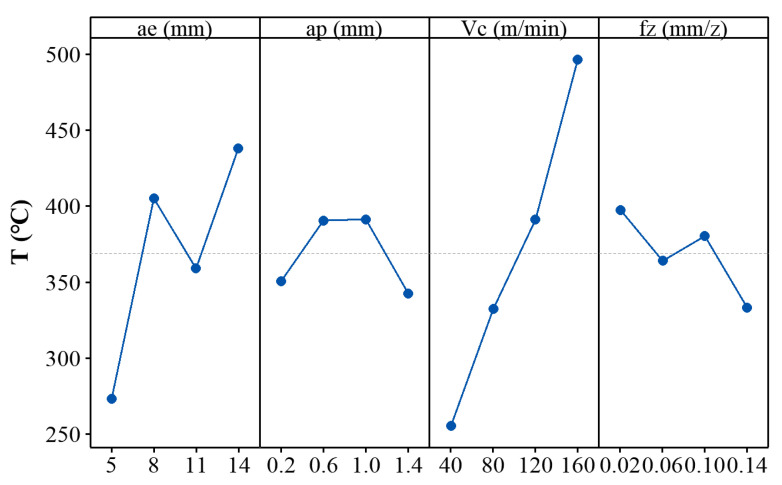
Plot of means for the maximum cutting temperature *T*.

Figure 7 shows the plot of means for the maximum cutting temperature *T* at the cutting zone. It can be found that both the radial depth of cut and cutting speed have a significant effect on the cutting temperature. This is because the radial depth of cut as well as cutting speed determines the strain rate during the milling process, which in turn controls the cutting temperature. However, the influences of the axial depth of cut and the feed per tooth on the cutting temperature are significantly weakened. This is because the axial depth of cut and the feed per tooth are relatively small, and the corresponding effects on strain rate are weakened.

**Figure 8:**
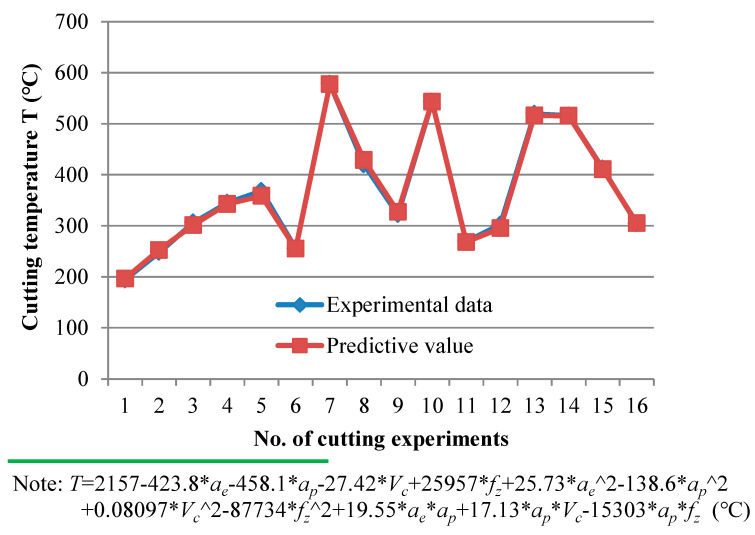
Experimental data [L_16_(4^4^)] and fitting results of maximum cutting temperature *T*.

Regression prediction model between cutting temperature and cutting parameters was established based on Taguchi analysis. The fitting results are shown in Figure 8 (the corresponding ANOVA results are summarized in Table 4), which shows that the use of the quadratic regression equation has strong predictability.

**Figure 9:**
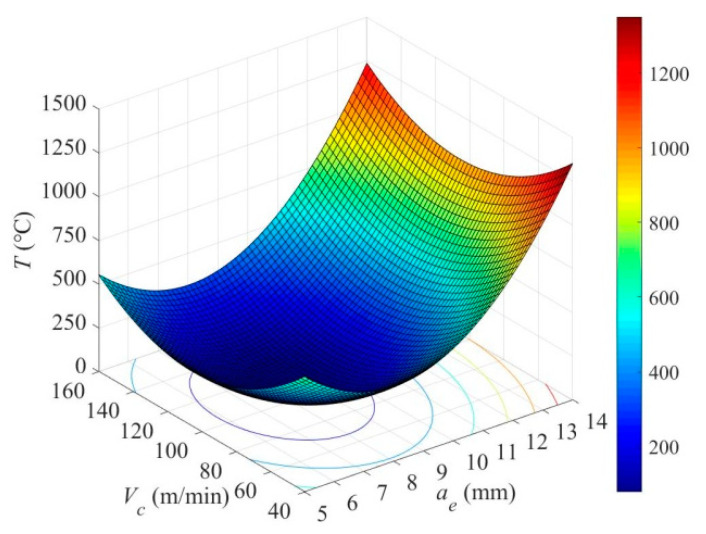
The interaction of *a_e_* × *V_c_* (*a_p_* = 0.6 mm, *f_z_* = 0.06 mm/z) on maximum cutting temperature *T*.

Figure 9 shows the interaction of radial depth of cut and cutting speed on cutting temperature. It can be seen from Figure 9 that the cutting temperature first decreases and then increases with the increase in radial depth of cut and cutting speed. The cutting temperature reaches the lowest level when the radial depth of cut is *a_e_* = 8 mm and the cutting speed is *V_c_* = 110 m/min. The reason can be accounted to the better cutting stability and heat dissipation under the fixed milling conditions. When the radial depth of cut or cutting speed is further increased, the adiabatic shearing is intensified, where the heat is concentrated. As a result, the temperature rises rapidly, even reaching above 1000 °C, in account with the adequate heat dissipation during the machining of titanium alloys. Therefore, the radial depth of cut and cutting speed should be optimized with the goal of minimizing the cutting temperature (i.e., the lowest point of the cloud chart in Figure 9).

### 3.3. The Effect of Cutting Parameters on Surface Roughness

**Figure 10:**
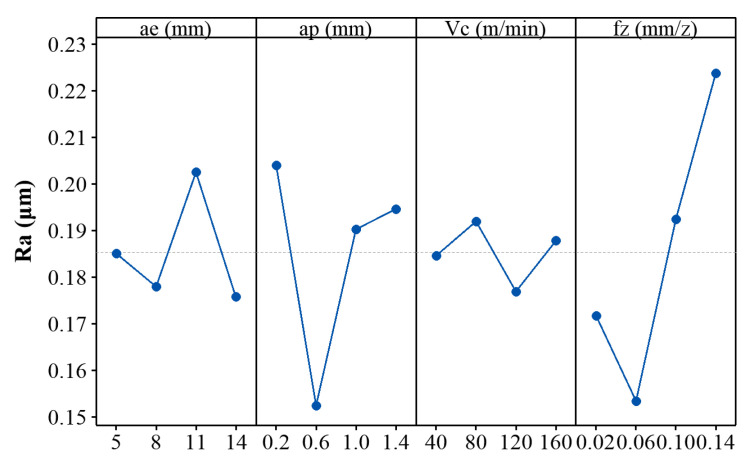
Plot of means for the machined surface roughness *R_a_*.

Figure 10 shows the plot of means for the machined surface roughness *R_a_*. It can be seen from Figure 10 that the feed per tooth has the most significant influence on the surface roughness. This can be explained from the perspective of cutting kinematics. As we all know, the feed per tooth and the tool nose are the key factors that determine the profile of the machined surface, which in turn determines the surface roughness value. Following with feed per tooth, the axial and radial depths of cut also generate considerable influence on the surface roughness. As mentioned in Section 3.1, the axial and radial depths of cut determine the magnitude and direction of the cutting force, which makes the milling tool holder subject to an unbalanced radial force during the asymmetric up-milling process, and in turn, causes deflection of the milling tool or cutting chatter, thereby changing the surface roughness. The cutting speed has the smallest effect on the surface roughness. This is because the surface generation mechanism in this speed range is always plastic removal and adiabatic shearing, and there is no mechanistic change.

**Figure 11:**
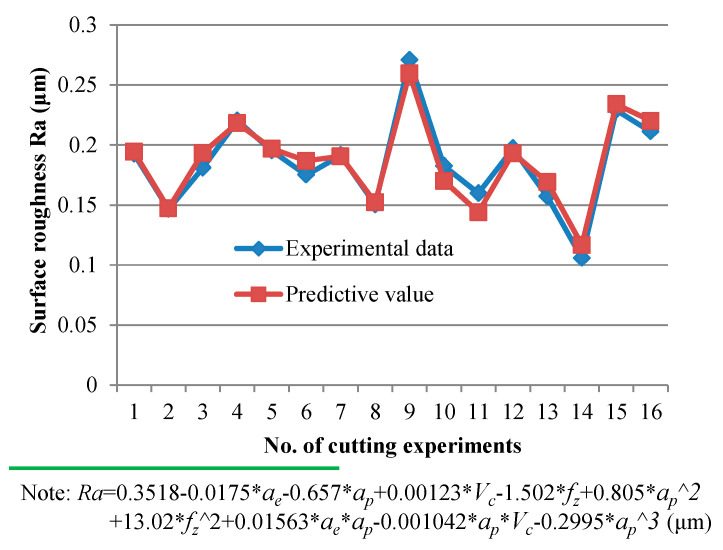
Experimental data [L_16_(4^4^)] and fitting results of surface roughness *R_a_*.

The regression prediction model between surface roughness and cutting parameters was also established based on Taguchi analysis. The fitting results are shown in Figure 11 (the corresponding ANOVA results are summarized in Table 5), which show that the use of the quadratic regression equation also has strong predictability on surface roughness.

**Figure 12:**
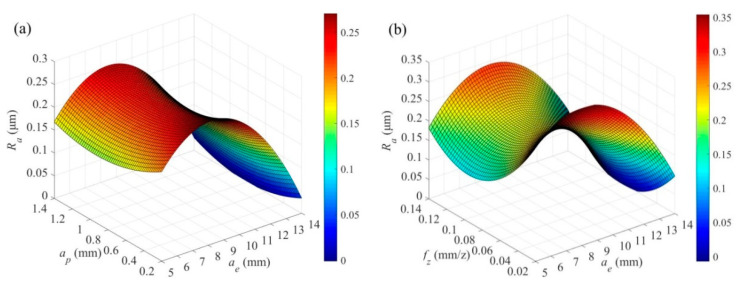
The interaction of (**a**) *a_e_* × *a_p_* (*V_c_* = 80 m/min, *f_z_* = 0.06 mm/z), and (**b**) *a_e_* × *f_z_* (*V_c_* = 80 m/min, *a_p_* = 0.6 mm) on surface roughness *R_a_*.

Figure 12a,b are the interactions of radial depth of cut versus axial depth of cut, and radial depth of cut versus feed per tooth on the surface roughness, respectively. It can be seen from Figure 12 that the surface roughness first increases and then decreases as the radial depth of cut increases. The surface roughness is the worst when the radial depth of cut is *a_e_* = 8 mm. This is because the feed marks are intertwined together as the radial depth of cut increases, and as a result, the surface roughness is deteriorated. However, the cutting temperature increases with the further increase in the radial depth of cut. Under this circumstance, a hard milling process makes the surface roughness decrease.

As shown in Figure 12, the surface roughness first decreases and then increases as the axial depth of cut and feed per tooth increase. The surface roughness reaches its best when the axial depth of cut is *a_p_* = 0.9 mm and the feed per tooth is *f_z_* = 0.09 mm/z. This is because the plough effect significantly deteriorates the surface roughness with small axial depth of cut or feed per tooth. The surface residue increases with the increase in the axial depth of cut and feed per tooth, that is, the surface roughness increases. Therefore, larger radial depth of cut, as well as optimized axial depth of cut and feed per tooth should be carefully selected in order to obtain the smallest surface roughness. However, the cutting force and cutting temperature are generally high under the cutting condition, which put forward higher requirements for tool tolerance.

### 3.4. The Effect of Cutting Parameters on Machinability

From the above analysis, contradictory results will be obtained when a different index is used for parameter optimization. In this section, radar chart is utilized for comprehensive machinability evaluation including the material removal rate MRR (i.e., Equation (1)), cutting force *F*, specific cutting energy *E_u_* (i.e., Equation (2)), cutting temperature *T*, and surface roughness *R_a_*, in order to optimize the cutting parameters in asymmetric up-milling of TC25.

(1) $$\mathrm{MRR}=a_{e}\times a_{p}\times f_{z}\times z\times \frac{1000\cdot V_{c}}{\pi D}$$

(2) $$E_{u}=\frac{F\cdot V_{c}}{\mathrm{MRR}}$$

The machinability could be evaluated based on the radar diagram method. In general, the evaluation value can be quantitatively expressed as the function of the area and perimeter of the radar diagram [29,30,31]. The steps of comprehensive machinability evaluation are briefly illustrated as follows:

1. Normalization treatment: In general, both the units and the numerical ranges are different for different machinability indexes, so it cannot be directly used for comprehensive machinability evaluation. Each index value should be first transformed into the interval of [0, 1] by normalization.
  (3) $${\bar{x}}_{ij}=\frac{x_{ij}-x_{i\min}}{x_{i\max}-x_{i\min}}$$
  where *x_i_*_min_ and *x_i_*_max_ represent the minimum and maximum value of the evaluation objective *x_i_*;
2. Standardization treatment: The beneficial index (the higher the index value, the better the machinability) and non-beneficial index (the higher the index value, the worse the machinability is) should be treated by standardization, separately, to ensure the same monotonicity. In the present study, all the cutting force, cutting temperature and surface roughness are non-beneficial indexes;
  (4) $$y_{ij}=\{\begin{array}{ll} 1-e^{-{\bar{x}}_{ij}}, & \mathrm{for}\;\mathrm{beneficial}\;\mathrm{index}\\ e^{-{\bar{x}}_{ij}}, & \mathrm{for}\;\mathrm{non}\text{-}\mathrm{beneficial}\;\mathrm{index} \end{array}$$
3. Extracting eigenvalues: Create the radar diagram, and extract the area (i.e., Equation (5)) and perimeter (i.e., Equation (6)) of the radar diagram as the eigenvalues (assuming *p*-th evaluation indexes of each evaluation object).
  (5) $$a_{i}=p\cdot \frac{\sum_{k=1}^{p-1}\sum_{h>k}^{p}\frac{1}{2}y_{ik}y_{ih}\sin\theta_{kh}}{C_{p}^{2}}$$
  (6) $$c_{i}=p\cdot \frac{\sum_{k=1}^{p-1}\sum_{h>k}^{p}\sqrt{y_{ik}^{2}+y_{ih}^{2}-2y_{ik}y_{ih}\cos\theta_{kh}}}{C_{p}^{2}}$$
4. Defining evaluation quantities:
  (7) $$v_{i1}=\frac{a_{i}}{a_{i\max}}$$
  (8) $$v_{i2}=\frac{a_{i}}{\pi \cdot {\left(\frac{c_{i}}{2\pi }\right)}^{2}}=\frac{4\pi a_{i}}{c_{i}^{2}}$$

The evaluation quantity *v_i_*_1_ reflects the relative size of the radar diagram, while the evaluation quantity *v_i_*_2_ reflects the balance of various indexes;

5. Evaluation results: Take the geometric mean of the two evaluation quantities as the evaluation results.
  (9) $$f\left(v_{i1},v_{i2}\right)=\sqrt{v_{i1}\cdot v_{i2}}$$

Table 6 shows the evaluation results of machinability.

**Figure 13:**
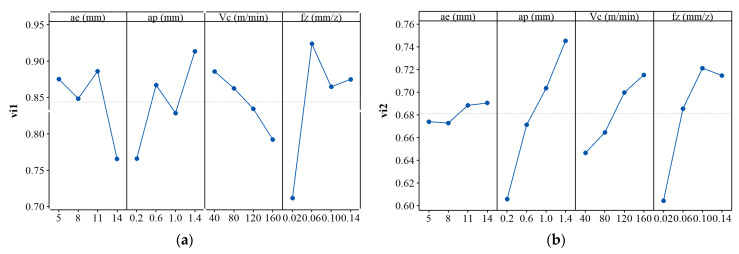

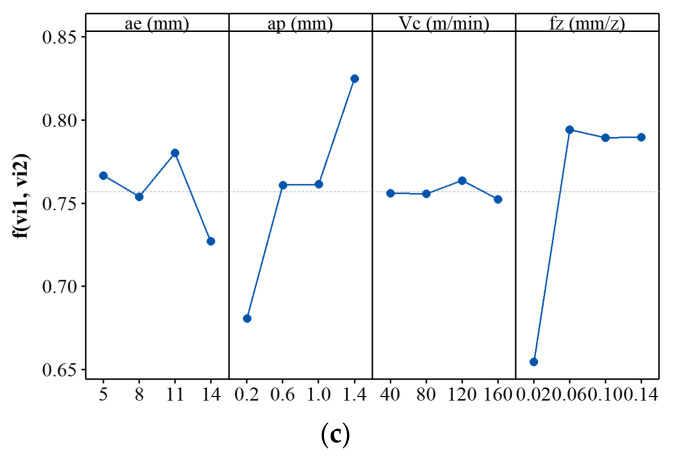
Plot of means for (**a**) evaluation quantity *v_i_*_1_, (**b**) *v_i_*_2_, and (**c**) machinability evaluation results *f*(*v_i_*_1_, *v_i_*_2_) in asymmetric up-milling TC25.

Figure 13 shows the plot of means for each evaluation quantity and the evaluation result of the machinability. Figure 13a shows that each cutting parameter has a significant impact on *v_i_*_1_. From the previous analysis, each cutting parameter will affect the evaluation indexes to different degrees, which are reflected as an impact on the area of radar diagram. Figure 13b shows that the influence of radial depth of cut on *v_i_*_2_ is significantly lower than other cutting parameters. It is indicated that a balanced development of the machinability can be reached by changing the radial depth of cut. Figure 13c shows that the machinability evaluation result is mainly affected by the radial and axial depths of cut, while the cutting speed is the least. This is mainly because the cutting speed has an opposite effect on the evolution of the area of radar diagram and the balance of various indexes.

**Figure 14:**
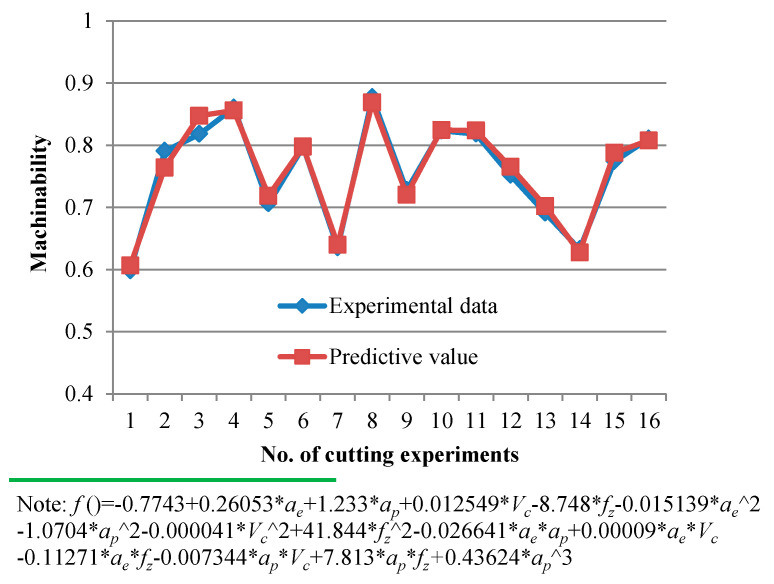
Comparison between the machinability evaluation results and fitting results.

The machinability evaluation results can be better predicted by establishing a quadratic regression model, as shown in Figure 14. The ANOVA results for machinability are summarized in Table 7.

**Figure 15:**
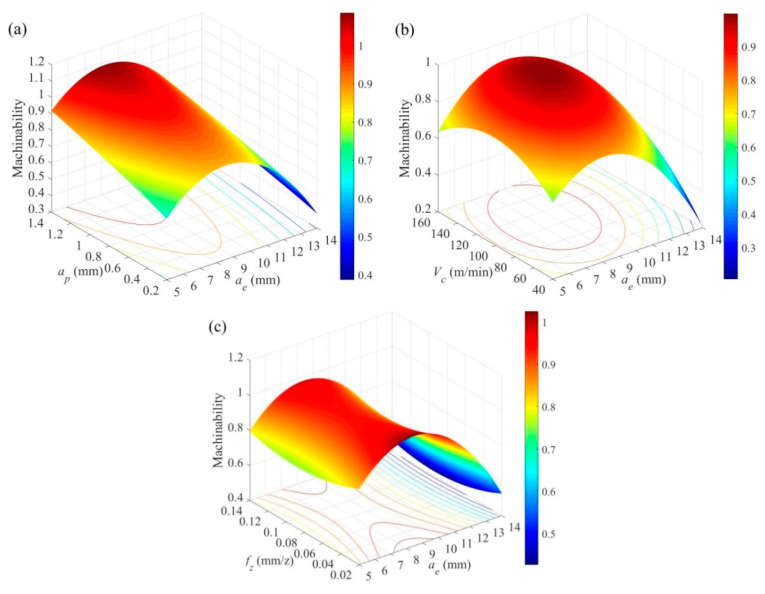
The interaction of (**a**) *a_e_* × *a_p_* (*V_c_* = 80 m/min, *f_z_* = 0.06 mm/z), (**b**) *a_e_* × *V_c_* (*a_p_* = 0.6 mm, *f_z_* = 0.06 mm/z), and (**c**) *a_e_* × *f_z_* (*V_c_* = 80 m/min, *a_p_* = 0.6 mm) on the comprehensive machinability.

The machinability in asymmetric up-milling TC25 is analyzed based on the regression model, as shown in Figure 15. It can be found that the machinability is first improved and then becomes weakened when the radial depth of cut is increased. This is the result of the comprehensive evaluation of cutting force/cutting temperature (first decreasing and then increasing) and surface roughness (first increasing and then decreasing). The machinability is improved as the axial depth of cut increasing. At the circumstance, the machinability is mainly affected by material removed rate in order to realize high-effective machining. The machinability first increases and then decreases as the cutting speed increases. The reason can be accounted to the heat dissipation conditions. The machinability first decreases and then increases with the feed per tooth increasing. This shows that TC11 titanium alloy is suitable for machining with a large feed rate to avoid work hardening.

**Figure 16:**
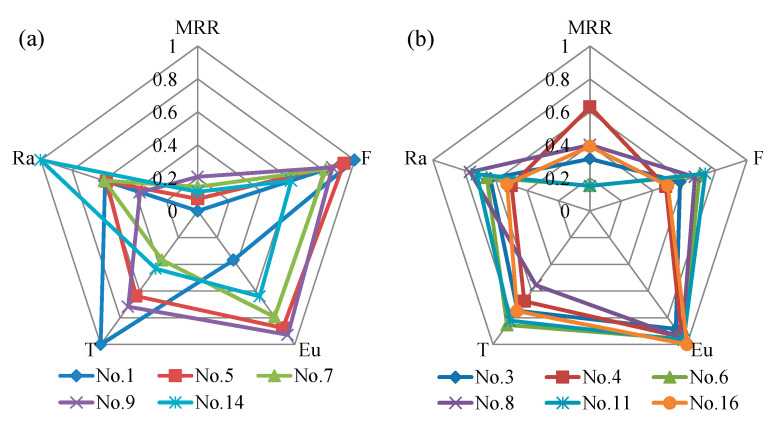
Typical radar diagrams for (**a**) bad, and (**b**) good machinability in asymmetric up-milling TC25.

The machinability evaluation results under certain machining conditions are shown in Figure 16. The poor balance of the evaluation indexes in Figure 16a leads to poor machinability. The main reason is that the material removal rate under the cutting parameters is relatively low, and the specific cutting energy consumption is also increased. The evaluation indexes in Figure 16b shows much more balance, i.e., high material removal rate, low specific energy consumption, as well as considerable and controllable cutting force, cutting temperature and surface roughness, which is conducive to the high effective machining of TC25.

## 4. Conclusions

Although the kinematics and advantages and disadvantages of the milling process are well known, the milling process of TC25 alloy still lacks theoretical guidance and limits the application of TC25 for aero engines and/or compressor parts on engines. In this paper, the evolutions of cutting force, cutting temperature, and surface roughness, and the corresponding machinability in asymmetric up-milling of TC25 alloy are investigated based on the Taguchi method. The key results and conclusions can be drawn as the following:

1. Asymmetric up-milling should be the first choice for milling of TC25 with respect to machinability. However, the radial depth of cut generates the opposite influence on the cutting force/cutting temperature versus surface roughness. The reason can be accounted as the intertwining of feed marks at a low radial depth of cut, and the mechanism of hard cutting at a high radial depth of cut. The optimized radial depth of cut cannot be obtained through a single analysis on each machinability index;
2. The machinability evaluation method based on a radar diagram is improved. The benefit of the improvement is that reliable eigenvalues can be obtained through this method. Moreover, different machinability indexes can be calculated together in order to evaluate the machinability. The results indicate that the asymmetry has a significant effect on the machinability in asymmetry up-milling of TC25 alloy. Changing the asymmetry, i.e., the radial depth of cut, can alter the machinability while maintain the balanced development of various indexes. The machinability reaches its best when radial depth of cut is *a_e_* = 8 mm;
3. The other cutting parameters also have a certain impact on the machinability. The axial depth of cut and feed per tooth should be selected as large as possible to avoid work hardening and to improve the machining efficiency in asymmetric up-milling TC25 alloy. The cutting speed should be controlled within *V_c_* = 100–120 m/min to obtain better machinability. On the basis of this research, the high-quality and high efficiency milling of TC25 alloy can be realized.

## Figures and Tables

**Table 1:** Factors and levels for orthogonal experimental design.

Level	Factor			
	Radial Depth of Cut *a_e_* (mm)	Axial Depth of Cut *a_p_* (mm)	Cutting Speed *V_c_* (m/min)	Feed per Tooth *f_z_* (mm/z)
−2	5	0.2	40	0.02
−1	8	0.6	80	0.06
+1	11	1	120	0.1
+2	14	1.4	160	0.14

**Table 2:** L_16_(4^4^) orthogonal series and experimental results.

No. of Trials	Radial Depth of Cut *a_e_* (mm)	Axial Depth of Cut *a_p_* (mm)	Cutting Speed *V_c_* (m/min)	Feed per Tooth *f_z_* (mm/z)	Experimental Results		
					Cutting Force *F* (N)	Cutting Temperature *T* (℃)	Surface Roughness *R_a_* (μm)
1	−2	−2	−2	−2	111.17	194.3	0.19
2	−2	−1	−1	−1	239.17	248.1	0.15
3	−2	+1	+1	+1	466.65	306.3	0.18
4	−2	+2	+2	+2	577.71	344.8	0.22
5	−1	−2	−1	+1	156.55	367.7	0.20
6	−1	−1	−2	+2	342.32	255.0	0.18
7	−1	+1	+2	−2	241.45	578.2	0.19
8	−1	+2	+1	−1	368.98	420.1	0.15
9	+1	−2	+1	+2	209.41	321.9	0.27
10	+1	−1	+2	+1	356.97	543.4	0.18
11	+1	+1	−2	−1	308.51	269.2	0.16
12	+1	+2	−1	−2	275.42	301.7	0.20
13	+2	−2	+2	−1	257.31	519.0	0.16
14	+2	−1	+1	−2	443.44	516.3	0.11
15	+2	+1	−1	+2	752.24	412.2	0.23
16	+2	+2	−2	+1	561.71	304.4	0.21

**Table 3:** ANOVA for maximum cutting force.

Parameters	DOF	Adj SS	Adj MS	*F*-Value	*p*-Value
Regression	7	427,900	61,128.5	67.47	0
*a_e_*	1	50,494	50,493.5	55.73	0
*a_p_*	1	9629	9628.8	10.63	0.012
*V_c_*	1	13,717	13,717.1	15.14	0.005
*f_z_*	1	97,896	97,895.7	108.05	0
a_e_*a_e_	1	82,626	82,626.1	91.19	0
a_p_*a_p_	1	25,003	25,002.7	27.6	0.001
a_p_*V_c_	1	19,048	19,048	21.02	0.002
Noise	8	7248	906.1	-	-
Total	15	435,148	-	-	-
*S*: 30.1008; *R*-sq.: 98.33%; *R*-sq. (adj): 96.88%; *R*-sq. (pred): 92.12%					

**Table 4:** ANOVA for maximum cutting temperature.

Parameters	DOF	Adj SS	Adj MS	*f*-Value	*p*-Value
Regression	11	206,102	18,736.6	263.18	0
*a_e_*	1	8133	8133.4	114.24	0
*a_p_*	1	8159	8158.7	114.6	0
*V_c_*	1	10,438	10,438	146.61	0
*f_z_*	1	11,971	11,970.8	168.14	0
a_e_*a_e_	1	8849	8848.6	124.29	0
a_p_*a_p_	1	7868	7867.7	110.51	0
V_c_*V_c_	1	10,741	10,741.2	150.87	0
f_z_*f_z_	1	14,331	14,331	201.3	0
a_e_*a_p_	1	4401	4401.1	61.82	0.001
a_p_*V_c_	1	14,303	14,302.8	200.9	0
a_p_*f_z_	1	9992	9991.9	140.35	0
Noise	4	285	71.2	-	-
Total	15	206,387	-	-	-
*S*: 8.43764; *R*-sq.: 99.86%; *R*-sq. (adj): 99.48%; *R*-sq. (pred): 98.33%					

**Table 5:** ANOVA for surface roughness.

Parameters	DOF	Adj SS	Adj MS	*f*-Value	*p*-Value
Regression	9	0.019946	0.002216	12.92	0.003
*a_e_*	1	0.003835	0.003835	22.36	0.003
*a_p_*	1	0.003983	0.003983	23.22	0.003
*V_c_*	1	0.002459	0.002459	14.34	0.009
*f_z_*	1	0.001912	0.001912	11.15	0.016
a_p_*a_p_	1	0.003274	0.003274	19.09	0.005
f_z_*f_z_	1	0.003788	0.003788	22.08	0.003
a_e_*a_p_	1	0.002813	0.002813	16.4	0.007
a_p_*V_c_	1	0.001333	0.001333	7.77	0.032
a_p_*a_p_*a_p_	1	0.002645	0.002645	15.42	0.008
Noise	6	0.001029	0.000172	-	-
Total	15	0.020975	-	-	-
*S*: 0.0131; *R*-sq.: 95.09%; *R*-sq. (adj): 87.73%; *R*-sq. (pred): 62.45%					

**Table 6:** Machinability evaluation indexes (original value and treated results by normalization and standardization) and the comprehensive evaluation values.

	MRR (mm^3^/min)		*F* (N)		*E_u_* (GN/m^2^)		*T* (℃)		*R_a_* (μm)		*a_i_*	*c_i_*	*v_i_*_1_	*v_i_*_2_	*f*
	ORI	TRE	ORI	TRE	ORI	TRE	ORI	TRE	ORI	TRE					
1	10.19	0.00	111.17	1.00	436.58	0.37	194.3	1.00	0.19	0.59	0.75	4.31	0.71	0.50	0.60
2	183.35	0.08	239.17	0.82	104.36	0.82	248.1	0.87	0.15	0.78	1.03	4.50	0.98	0.64	0.79
3	763.94	0.32	466.65	0.57	73.31	0.88	306.3	0.75	0.18	0.63	0.93	3.91	0.88	0.76	0.82
4	1996.44	0.63	577.71	0.48	46.29	0.94	344.8	0.68	0.22	0.50	0.98	3.93	0.93	0.79	0.86
5	162.97	0.07	156.55	0.93	76.84	0.88	367.7	0.64	0.20	0.58	0.86	4.21	0.82	0.61	0.71
6	342.25	0.15	342.32	0.70	40.01	0.96	255.0	0.85	0.18	0.66	1.00	4.35	0.95	0.67	0.80
7	325.95	0.15	241.45	0.82	118.52	0.79	578.2	0.38	0.19	0.60	0.66	3.62	0.63	0.64	0.64
8	1026.74	0.40	368.98	0.67	43.12	0.95	420.1	0.56	0.15	0.76	1.04	4.10	0.99	0.78	0.88
9	470.59	0.21	209.41	0.86	53.41	0.93	321.9	0.72	0.27	0.37	0.85	4.06	0.81	0.65	0.73
10	1344.54	0.49	356.97	0.68	42.48	0.95	543.4	0.40	0.18	0.63	0.93	3.89	0.88	0.77	0.82
11	336.14	0.15	308.51	0.74	36.71	0.97	269.2	0.82	0.16	0.72	1.05	4.44	1.00	0.67	0.82
12	313.73	0.14	275.42	0.77	70.23	0.89	301.7	0.76	0.20	0.58	0.90	4.12	0.85	0.66	0.75
13	342.25	0.15	257.31	0.80	120.29	0.79	519.0	0.43	0.16	0.73	0.76	3.82	0.73	0.66	0.69
14	256.69	0.12	443.44	0.60	207.31	0.64	516.3	0.43	0.11	1.00	0.69	3.76	0.65	0.61	0.63
15	1996.44	0.63	752.24	0.37	30.14	0.98	412.2	0.57	0.23	0.47	0.84	3.77	0.80	0.75	0.77
16	998.22	0.39	561.71	0.50	22.51	1.00	304.4	0.75	0.21	0.53	0.93	3.95	0.88	0.75	0.81

**Table 7:** ANOVA for machinability.

Parameters	DOF	Adj SS	Adj MS	*f*-Value	*p*-Value
Regression	14	0.105499	0.007536	9310.72	0.008
*a_e_*	1	0.003024	0.003024	3736.21	0.01
*a_p_*	1	0.00456	0.00456	5634.38	0.008
*V_c_*	1	0.002136	0.002136	2639.55	0.012
*f_z_*	1	0.001317	0.001317	1627.67	0.016
a_e_*a_e_	1	0.003062	0.003062	3783.41	0.01
a_p_*a_p_	1	0.002356	0.002356	2910.79	0.012
V_c_*V_c_	1	0.002704	0.002704	3340.94	0.011
f_z_*f_z_	1	0.00041	0.00041	506.86	0.028
a_e_*a_p_	1	0.002233	0.002233	2758.79	0.012
a_e_*V_c_	1	0.000319	0.000319	394.09	0.032
a_e_*f_z_	1	0.000908	0.000908	1122.03	0.019
a_p_*V_c_	1	0.00263	0.00263	3249.22	0.011
a_p_*f_z_	1	0.002604	0.002604	3217.59	0.011
a_p_*a_p_*a_p_	1	0.002265	0.002265	2798.77	0.012
Noise	1	0.000001	0.000001	-	-
Total	15	0.1055	-	-	-
*S*: 0.0008996; *R*-sq.: 100.00%; *R*-sq. (adj): 99.99%; *R*-sq. (pred): 99.01%					

## Data Availability

Data sharing is not applicable.
